# COVID-19 Vaccination Perceptions Among Young Adults of Color in the San Francisco Bay Area

**DOI:** 10.1089/heq.2022.0068

**Published:** 2022-11-10

**Authors:** Francine Rios-Fetchko, Mariam Carson, Mario Gonzalez Ramirez, Jonathan Z. Butler, Roberto Vargas, Abby Cabrera, Angela Gallegos-Castillo, Monique LeSarre, Michael Liao, Kent Woo, Randi Ellis, Kirsten Liu, Brittney Doyle, Lydia Leung, Kevin Grumbach, Alicia Fernandez

**Affiliations:** ^1^ Department of Medicine, UCSF Latinx Center of Excellence, University of California, San Francisco, San Francisco, California, USA.; ^2^ Department of Family and Community Medicine, University of California, San Francisco, San Francisco, California, USA.; ^3^ Stanford University, Stanford, California, USA.; ^4^ Instituto Familiar de la Raza, Inc., San Francisco, California, USA.; ^5^ Rafiki Coalition for Health and Wellness, San Francisco, California, USA.; ^6^ NICOS Chinese Health Coalition, San Francisco, California, USA.; ^7^ Department of Medicine, UCSF Black Health Initiative, University of California, San Francisco, San Francisco, California, USA.

**Keywords:** COVID-19 vaccination, young adults, vaccine hesitancy, disparities

## Abstract

**Background:**

COVID-19 vaccination rates among U.S. young adults, particularly in communities of color, remain lower than other age groups. We conducted a qualitative, community-based participatory study to explore beliefs and attitudes about COVID-19 vaccines among young adults in Black/African American, Latinx, and Asian American or Pacific Islander (AAPI) communities in the San Francisco Bay Area.

**Methods:**

We conducted six focus groups between June and August 2021. Participants were recruited by partnering with community-based organizations in the San Francisco Bay Area. Focus groups included Black/African American (*N*=13), Latinx (*N*=20), and AAPI (*N*=12) participants between 18 and 30 years of age. Emerging themes were identified using a modified Grounded Theory approach.

**Results:**

Prominent themes among all three racial-ethnic groups included mistrust in medical and government institutions, strong conviction about self-agency in health decision-making, and exposure to a thicket of contradictory information and misinformation in social media. Social benefit and a sense of familial and societal responsibility were often mentioned as reasons to get vaccinated. Young adult mistrust had a generational flavor fueled by anger about increasing inequity, the profit-orientation of pharmaceutical companies and health institutions, society's failure to rectify injustice, and pessimism about life prospects.

**Conclusion:**

Factors influencing vaccine readiness among Black/African American, Latinx, and AAPI young adults have a distinct generational and life-course texture. Outreach efforts should appeal to young adults' interest in family and social responsibility and the social benefits of vaccination, while being cognizant of the friction mandates pose for young adults' sense of self-agency. Efforts will be most effective coming from trusted messengers with a proven commitment to communities of color and health equity.

## Introduction

Vaccines against SARS-CoV-2 are safe and effective, and significantly reduce the risk of transmission, hospitalization, and death due to COVID-19.^1–4^ However, vaccination rates have plateaued in the United States, with 69% of the population fully vaccinated as of mid-November 2021, when this study was completed.^5^

COVID-19 vaccine uptake is lower among adults aged 18–24 than among older age groups.^6,7^ National vaccination rates disaggregated by both age and race-ethnicity are not readily available, but COVID-19 vaccination rates are lowest among African American and Asian American or Pacific Islander (AAPI) populations for the overall eligible U.S. population.^8,9^

Lack of vaccine readiness during the pandemic has been well documented, especially among adults in communities of color.^10–13^ Mistrust in government and health/research institutions, as well as misinformation and disinformation perpetuated by news and social media, are frequently cited as barriers to uptake.^10^ While communities of color have been surveyed about their beliefs and attitudes toward the COVID-19 vaccines, little research on vaccine confidence has specifically studied young adults of color.^14,15^ A life course perspective may be helpful for understanding lower vaccine uptake among young adults. Age-related factors such as differences in risk of severe illness from COVID-19, developmental stage of transitioning from adolescence to adulthood, and generation-based perspectives and experiences may influence the views of young adults.^16,17^

We conducted a qualitative study to investigate the beliefs and attitudes toward COVID-19 vaccines among Black/African American, Latinx, and AAPI young adults. We used principles of community-based participatory research to develop and execute this study, with the goal of generating new knowledge that could be immediately used by community partners and other stakeholders to inform vaccine outreach efforts.

## Methods

Three community-based organizations (CBOs) partnered with university-based researchers in all phases of this focus group study, serving as coinvestigators and collaborators in the development of focus group guides, participant recruitment, data analysis, and dissemination of findings. The CBOs—Rafiki Coalition, Instituto Familiar de la Raza and NICOS Chinese Health Coalition—served as first point of contact with study participants. Research was approved by the University of California, San Francisco (UCSF) Institutional Review Board (Study No. 20-32672).

### Recruitment

To understand diverse perspectives about the COVID-19 vaccines, we aimed to recruit both vaccinated and unvaccinated participants with an emphasis on the latter. Local CBOs offering services and programs to low-income young adults from the race/ethnic populations of interest recruited participants through phone calls, emails, and fliers to their clientele. Inclusion criteria included self-identifying as Black or African American, Latinx, or AAPI, fluency in English, residence in the San Francisco Bay Area, and age 18–30 years old. For the AAPI community, we emphasized recruitment of self-identifying Pacific Islander young adults, as input from community partners highlighted lower vaccination rates and high need in this community. Over half of our AAPI participants identified as Pacific Islander. Consent was obtained verbally over the phone. Participants completed a short questionnaire about demographic and household information before the focus groups. Both the questionnaire and consent were administered by a UCSF research analyst.

### Data collection

Six focus groups were held between June and August 2021. Focus groups were organized by race-ethnicity, except for one focus group containing a mixture of participants from all racial-ethnic groups to facilitate a cross-ethnic discussion. Focus groups of 6–10 participants were conducted over Zoom for 60–75 min, cofacilitated by university and CBO researchers. Zoom sessions were recorded and transcribed. Participants were compensated with $100 gift cards.

The focus group guide began with general questions about COVID-19 pandemic perceptions and impacts and then asked open-ended questions about COVID-19 vaccine beliefs and attitudes (see Appendix A1 for interview guide).

### Data analysis

Three research analysts identified emerging themes from the transcripts using a modified Grounded Theory approach.^18^ These themes were used to generate a codebook using Dedoose, a qualitative coding software, which was then refined through discussions identifying and categorizing themes with the entire research study team, including CBO partners, and applied to each transcript.

## Results

Forty-six young adults participated in the study (Table 1). Several themes emerged as influencing participants' reluctance to or decision not to be vaccinated (Table 2). These included mistrust of the government and/or medical institutions, a strong desire for autonomy over health care decision-making, and high volumes of misinformation.

**Table 1.** Characteristics and Vaccination Status of COVID-19 Vaccine Perception Study Participants

	Total	African American	Latinx	AAPI
Total	45	13	20	12
Gender, female	27	7	12	8
Gender, male	18	6	8	4
Currently working outside home yes	27	6	11	10
Currently working outside home no	18	7	9	2
serious chronic health condition, yes	9	0	7	2
serious chronic health condition, no	36	13	13	10
Num of ppl in hh, 1–2	13	3	7	3
Num of ppl in hh, 3–4	17	6	9	2
Num of ppl in hh, 5 or more	15	4	4	7
Flu shot, yes	15	6	5	4
Flu shot, no	30	7	15	8
Fully vaccinated against COVID-19	14	9	2	3
Not vaccinated against COVID-19	19	2	11	6
Received first vaccine dose and does not plan to receive second dose	1	1	0	0
Did not disclose vaccination status	12	2	7	3

*Note:* One of the 46 study participants did not complete the questionnaire and therefore has missing data for table items.

AAPI, Asian American or Pacific Islander.

**Table 2.** Key themes and illustrative quotes

Theme	Quote
Mistrust	
Historical injustice	I know it's also been talked about very heavily in just communities of color, all different races saying that there's a lot of [push] back from people trying to get the vaccine just because like historically you know, vaccines and things of that nature have been tested on like people of color and it's just—it's been rather scary and it's something that's been recorded in the history books, so I'm sure that's the biggest word that I've heard as to why people don't wish to get the vaccine. Just general fear of things that have already been happened. (Woman, Latinx)
Contemporary neglect	It made me very much question like if you guys are over here pushing back on giving us healthcare, how is it that you are able to mass produce these vaccines and make them available for free to everybody? (Woman, Latinx)
Personal negative experience with health care system	She just doesn't trust the health system just because they have neglected her before (Woman, Latinx)
Autonomy	
Mandates as an infringement on personal autonomy	They did some type of raffle or something where everyone that got vaccinated, and people won, like, a million dollars or something crazy like that. I don't know exact numbers, but I just feel like it's just—I just feel like there should be more facts instead of trying to force and incentive people to get it (Woman, Latinx) For me personally, I've never tried to convince anybody to get the vaccination, that's honestly a personal preference that you feel, and that you need to make your own personal self, to not anything I want to force on anybody else (Man, AAPI)
Resistant to pressure from health care system	I shouldn't be pressured into it. Other people shouldn't be forced to do it, especially if we're not at risk. (Woman, Latinx) I think that it's necessary for us to be able to, A, have the choice to not take it and, B, to like really know that we have our own health, is ours to trust, and it's like it's something that we do have agency over… I just think we have the right to be able to have these questions because it's very questionable where these things are coming from and we have a horrible track record of actually being able—of this state servicing for us in the right way. (Woman, African-American)
Misinformation	
Social media is predominant source of information despite knowing it is rife with misinformation	So, the misinformation a lot of the time is through social media, which is kind of funny, because for me, that's like a big place where I get news and what's going on in the world, so it depends. There's a lot of accounts out there that spread this misinformation about vaccines and conspiracy theories which of course it's okay to explore, but at times there is sometimes like misinformation that get put on to social media that a lot of people tend to believe. (Man, Latinx)
Motivators	
To protect family	I got it because of my mom, and also, she doesn't want to get it so, I'm like, okay, what is my duty to protect her? (Woman, Latinx)
Social and economic benefits	The only benefit to it is that the world is opening back up again, because everybody is getting. And I want to live my life outside of the house and so they're claiming that the more people get vaccinated the more things is going to open. (Woman, African-American)
Vaccine mandate	My boss came in one day and pretty much told us…they were really pushing us to get vaccinated because they didn't want us to catch the COVID from the homeless people that we were helping. So, yeah, they pretty much made us get ours. So that's the only reason why I got [vaccinated], to be honest. (Woman, Latinx)
Social and global responsibility	There are people waiting for it, and I think that that was what was most important to me, the fact that so many people were asking for it and waiting for it that I decided that I was going to take advantage of my privilege (Woman, Latinx) As a younger, relatively healthy individual, a motivator for me was just that fact that it is within my power to get vaccinated. And if it is within my power to do so, then I will do it as my part in the bigger picture. So just knowing that, like, as a personal motivator that you are kind of one more piece in the puzzle to help everybody move forward in this is a motivator for me. (Woman, Latinx)

### Mistrust

Mistrust was consistently stated as one of the reasons participants did not want to receive a COVID-19 vaccine. Participants expressed mistrust toward the government, medical institutions, and the vaccine itself. They attributed this mistrust to historical injustices, current disregard by the government, and negative personal experiences with institutions promoting the vaccine.

#### Mistrust based in history

For some, mistrust was rooted in historical injustices carried out by the government. One participant explained:

It's hard to really trust what's going on just because of the history of how these things were used against us Black and Brown people, particularly Black people, so it's like no wonder we're going to have trust issues related to this random vaccine created by a huge pharmaceutical company… (Woman, African-American)

Another participant echoed this sentiment, stating that “I'm not a lab rat. We aren't lab rats.” (Woman, African-American)

#### Mistrust based in contemporary neglect by the government

While some pointed to history as a source of mistrust, others cited current neglect by the government as the root of their skepticism. One participant summarized her sentiments around allocation of resources as follows:

Like you're putting all this money towards this shot that can harm us, but you're not going to put it towards things that can help us, kind of sketchy. (Woman, African-American)

For some, vaccine incentives were also seen within the context of contemporary neglect, leading one young adult to explain:

I think the incentives definitely make me more hesitant to want to get the vaccine. Something that I've seen other people bring up is, okay, well, people have cancer. They have asthma. They have all these other conditions. You don't see them giving out free treatment for that or begging you to get treatment for that stuff, so I think it is a little suspicious that they're pushing it so hard. Like, what's in this, you know? (Woman, AAPI)

Another pointed to lack of access to health care as a reason to question vaccine incentives, highlighting inconsistencies in the government's intentions:

So, I don't think that it should be forced down people's throats, especially if healthcare isn't being made widely accessible to everybody in our society. And I also think about where this money is coming from, where [are] the benefits and where is the profit that is being made from this. Where is it coming from? Where is it going? And then we're trusting these medical institutions, these big pharmaceutical institutions that have poisoned us to entrust them with our health. (Woman, Latinx)

These sentiments of mistrust—both historical and current—have cultivated a deep “us vs. them” divide between governmental and medical institutions promoting the vaccine and many study participants. One participant clearly expressed this view:

There is no, ‘we are in this together,’ it's them versus us. We can have community, but if people come in around and actually participate and take responsibility and give us resources and all this stuff, like definitely, absolutely. But I just don't think that the government would promise us that at all. I have no trust, no, nothing is enough. (Woman, African American)

The above quote highlights how mistrust manifested as pessimism about government support. Others expressed how their future plans were impacted, especially for young adults entering and navigating independence for the first time:

I think for me I was transitioning into a young adult and there was this like belief in my family that at some point I was supposed to achieve independence though this age group and COVID just made it a lot more difficult than it should have been if that makes sense. Typically, I would have been able to keep the jobs that I have or like moved up in the organization that I was in but because of COVID, the program that I was working were shut down. Everything around me was shutting down and I just had no next step option… (Woman, Latinx)

#### Mistrust fueled by personal negative experiences

Some participants mentioned personal or family experiences with medical institutions as a driver of their mistrust. One participant explained:

[My mom] had a heart attack and when she went to the doctor, they just told her it was like gastritis, like something with her stomach and then, two days later, she ended up having a heart attack. And so, ever since then, she just doesn't trust the health system. (Woman, Latinx)

#### Critique of profit motives

Criticism of the government and medical institutions—mainly pharmaceutical companies—was often driven by a view of American society as being “profit driven.” Comments about money and profit from the vaccines were common; one participant explained that vaccines and government “are all for themselves. …It's all about money, money, money, money, money.” (Woman, African-American)

### A focus on personal autonomy

Participants explicitly articulated a desire for self-determination and autonomy in medical and other decision-making. Many participants had negative reactions not only to family and friends urging them to be vaccinated, but to policies that required them to be vaccinated to engage in certain activities.

Why are you trying to vaccinate and then restricting me from how I move in the world. So now I can't travel because I don't have a vaccine? Now I can't work here because I don't have a vaccine? Because last time I checked that was a choice so I think it's very weird how they're forcing it on us. (Woman, African-American)

One participant in particular decided not to receive the second dose after receiving the first, explaining that a breach of her autonomy led to this decision:

I feel highly irritated at the fact that I allowed others to persuade my mind, to persuade my decision based off that push, right? That forcefulness, that harassing, it was too much. But I had to think about it like, ‘Do I really want to get the vaccine because somebody else is forcing me to do it or is this because I want to do it?’ So, I didn't go back because I was like, ‘Okay, well you all got me once, but I'm not going again.’ (Woman, African-American)

### Misinformation

The third driver of vaccine resistance among participants was misinformation. Participants said they encounter a lot of information, some of which they knew to be false and other statements that they simply could not evaluate as true or not, leaving them confused and feeding into a general feeling of mistrust. As one explained with regard to conflicting facts, youth “don't have the time, going to college, in school and what not, to sit down with Anthony Fauci [and] watch TV. We're on the go, or on our phone and we will watch it that way.” (Man, Latinx)

Social media played an especially influential role in disseminating misinformation. Despite recognizing that social media was unreliable, participants consistently cited social media as their go-to information source. One participant acknowledged the tension around social media conflation of potential vaccine side effects, explaining:

Everybody I know that got it, they're okay, but I just think everything I see on social media or whatever, is scary—it scares you away from getting it kind of because you hear about people, like, getting strokes in their face and the heart inflammation. (Woman, multi-ethnic FG)

Another participant explained how their younger relatives, also using social media, were unable to distinguish between misinformation and the truth:

A lot of them are like I've heard microchips, I've heard zombie apocalypse, I've heard a lot of different things, a lot of it is coming from my younger cousins and I noticed that some of them actually believe it and be telling people it's the truth when it's like okay, come on now, like kind of get some science behind that before you believe it. (Woman, AAPI)

Participants frequently cited the comments section of social media platforms as a reference tool to check the accuracy of a post.

Even for those that claimed to be able to sift through social media to find accurate information, determining the accuracy of some claims on the internet was still challenging. This was highlighted by various participants with the same specific misconception:

I also heard—I don't know if it was true or not, but I don't know what vaccine it was, but I guess wherever you got your vaccine needle pushed in, you became magnetic in that area. I don't know. I don't know if it was, like, some rumor or anything that was being passed on. (Man, AAPI)

### Motivators for getting vaccinated

Despite overall mistrust of the vaccine, participants pointed to the health of their family, social benefits, and societal reasons to get vaccinated.

#### Responsibility to family health

Participants were more concerned about the health of their parents and elderly family members than threats to their own health from COVID-19, and many felt motivated to receive the vaccine “to keep whoever safe that's in their household that may benefit.” (Woman, African-American)

#### Social and economic mandates

For many participants who had been vaccinated, perceived social or economic necessity motivated their decision. Participants referenced societal benefits of vaccination, including travel, attending sporting events, and entering restaurants and bars. For others, the main motivator for vaccine uptake was the ability to return to work in the face of workplace vaccine mandates. One participant explained how mandates affected her decision:

The school districts are making it mandatory for teachers and everybody that's in there to get vaccines, so that means if we are going to be partnering with them, we're going to have to get vaccinated too…It's kind of just making it feel like if you want to go back to a sense of normalcy, you're going to have to get the vaccine. If not, you're going to be very, very limited. (Man, AAPI)

#### Societal and global responsibility

Some participants expressed a sense of social responsibility as a motivation to get vaccinated. As one participant explained, “there's so many people in our community that are literally begging for this opportunity, like literally begging their case managers, begging people to be able to be vaccinated because whoever they talk to, whatever they think, they think that this is going to be safer.” (Woman, Latinx)

Discussions of social responsibility extended beyond participants' family and local communities. One participant “understood the value of what it meant to be on the frontlines of being able to get this vaccine before most people could,” (Woman, Latinx) expressing an understanding of themselves as a global citizen in a privileged position. One young woman reflected on what holding this privilege meant to her: “I was like I need to be able to take it simply because there are many people who don't have this opportunity and it would be selfish of me not to,” (Woman, Latinx) in reference to the lack of access to the vaccine in countries such as Mexico, India, and Brazil.

## Discussion

Prominent themes that emerged from our focus groups with African American/Black, Latinx, and AAPI young adults as explanations for reluctance to get a COVID-19 vaccine included deep-rooted mistrust in medical and government institutions, a strong conviction around personal agency in health decision-making, and a thicket of contradictory information and misinformation. Social benefit and a sense of familial and societal responsibility featured prominently as reasons to get vaccinated.

Although the views of young adults in our study overlapped in many ways with themes reported from studies of older adults from Black/African American, Latinx, and AAPI communities, there were some distinct features to the perspectives of young adults—including in comparison with the views expressed by older adults in similar communities in San Francisco.^10,19^ Our results are consistent with current findings that mistrust expressed by young adults is predominantly situated in contemporary issues and events, such as under-resourcing of communities of color and racist violence and neglect, rather than in historical narratives about medical abuse such as the Tuskegee syphilis study.^20^ Young adult mistrust was more all-encompassing than that of many older adults, fueled by anger about increasing inequality, the profit-orientation of pharmaceutical companies and health care institutions, and society's failure to rectify injustice.

Some participants expressed pessimism about overall life prospects for themselves and their generation, leading them to question the relevance of vaccines in the context of the looming issues affecting their future. A counterpoint to this pessimism was the sense of social responsibility and solidarity that several participants expressed as motivators for vaccination.

Participants overwhelmingly referenced social media as their main source of information. Many acknowledged that social media was unreliable and filled with misinformation about COVID-19 vaccines, yet, still engaged with these platforms over traditional news outlets. The pervasiveness of social media in young adult lives is especially important to consider when developing messaging strategies to target this group. Leveraging social media as an educational platform has potential to reach younger audiences; however, without significant investment of resources, these messages may be swallowed by the vast sea of information, misinformation, and disinformation that already lives on major platforms.

Sentiments about the importance of autonomy in decision-making about vaccines were consistently and emphatically voiced by young adult participants. Although U.S. individuals of all ages tend to highly value self-determination, affirming independence and self-agency is particularly salient for individuals at the developmental stage of early adulthood.^21^ The strong desire for autonomy contributed to criticism of “carrots and sticks” policies to induce people to get vaccinated. Several participants perceived financial incentives for vaccination as being manipulative, furthering their degree of mistrust. Other studies and news accounts have also found that financial incentives may be counterproductive for vaccine uptake.^22,23^ Participants were also overwhelmingly opposed to vaccination mandates. However, many participants who had been vaccinated cited workplace or school mandates as the reason for receiving their vaccine, suggesting that the social benefit of vaccination outweighed the loss of autonomy associated with compliance.

It is noteworthy that participants' expressions of autonomy with regard to vaccination differed from their views about other safety practices, such as mask wearing and social distancing. All participants viewed masking as a way to take control of their own health that respected their desire for self-determination, and were supportive of masking mandates, suggesting that opposition to vaccine mandates was less rooted in a libertarian ideal than in the intersection of preserving personal health care decision-making, medical mistrust, and navigating social and economic independence as young adults.

Our findings are consistent with existing literature on vaccine readiness among communities of color. Vaccine readiness is characterized as a spectrum, with individuals moving from areas of vaccine skepticism to vaccine confidence, based on new information and experiences that shape their individual decision-making processes.^24^ While some individuals in our focus groups were vaccinated and others dedicated to remaining unvaccinated, several participants used a “wait and see” approach to their vaccine decision-making. Similar to our findings, previous studies referenced historical injustices inflicted upon communities of color by medical/research institutions and the government as sources of mistrust.^10,15^ One study specifically highlighted participants' concerns around pharmaceutical profit motives.^10^

While other studies have evaluated young adults' intentions on getting vaccinated, they did not specifically focus on young adults in communities of color and often based their recruitment on text messaging and surveys, rather than collaborative community-driven recruitment and in-depth qualitative analysis.^13,14^

Based on our findings, we have three recommendations for public health departments, health care entities and community organizations to increase vaccine uptake in young adults of color: acknowledge the connection for many between vaccine discussion and broader concerns of social injustice; emphasize economic and social benefits of vaccination; and involve young social media influencers and young adult voices in information dissemination efforts (Table 3).

**Table 3.** Recommendations for Increasing Vaccine Uptake in Young Adults of Color

Recommendation	Description
1. Acknowledge and address the connection between vaccine discussions and broader concerns of social justice	Health care professionals should be prepared to engage in conversations around social injustices and acknowledge governmental failures when discussing the vaccine with their patients. Messengers from organizations with a demonstrated commitment toward equity and social investment may be best received by young adults. Conversations should be facilitated by those with a demonstrated commitment toward social justice, and can be held via community forums, events, and outreach programs. Messages should speak to young adults' sentiments of social and familial responsibility
2. Emphasize nonhealth benefits of vaccination. E.g. Social and economic benefits	Young adults expressed concerns about protecting elderly family members. They were also highly motivated by being able to return to work and engage in social activities. These benefits should be highlighted in public health messaging
3. Include young adult voices in information dissemination efforts. E.g. Through social media outreach and messaging campaigns	Young adults are best positioned to know how to reach and influence their peers. Public health campaigns should engage with and invest in leaders from this generation to begin to tackle issues of deep-rooted mistrust in preparation for future public health emergencies. Examples of outreach campaigns include sponsoring and amplifying provaccination social media influencers who are generationally relevant. Social media will continue to be the most prominent source of information for this age group, so there is a need to counter the overwhelming volume of misinformation with easily accessible, scientifically backed, reliable sources that young adults recognize and trust

Our study was limited to young adults from three race-ethnic groups living in the SF Bay Area who spoke English fluently and were able to engage over Zoom. Our findings may not generalize to other young adults or other racial-ethnic groups not included in this study. We did not have sufficient sample size to separately analyze responses from AAPI participants. Other studies have demonstrated a divergence between AAPI groups in vaccination rates and vaccination perspectives. The focus groups were conducted between June and August, 2021, coinciding with changes in vaccine eligibility criteria, Centers for Disease Control and Prevention recommendations, and an increase in local vaccine mandates.^25^

In summary, factors of mistrust, misinformation, and regard for personal autonomy that influence COVID-19 vaccine readiness among Black/African American, Latinx, and AAPI young adults have a distinct generational and life-course texture. Outreach efforts should take these factors into account and proceed accordingly by appealing to young adults' interest in family and social responsibility and the benefits of vaccination for travel, employment, and recreational activities while being cognizant of the friction mandates pose for young adults' interest in self-agency.

## Abbreviations Used

AAPI: Asian American or Pacific Islander
CBOs: community-based organizations
UCSF: University of California, San Francisco

## Appendix

### Appendix A1. Introduction

Thank you for taking the time to participate in this interview. My name is (introduce the interviewer).

As a reminder, we will keep the information we collect from you confidential, and we will not share your name or contact information with anyone outside the research team. As we discussed in the consent, we talked about recording this interview. If this is still okay with you, I will start recording our interview now.

1. **How has COVID-19 impacted you?**
Probes: or your family and friends; do you know anyone who has had COVID-19?
2. **Perceived susceptibility/severity, Health motivation**:
a. Do you try to protect yourself from COVID-19? If so, how do you try to protect yourself?
b. Who do you believe is at high-risk for COVID-19?
3. **Perceived knowledge**:
a. What, if anything, have you or members from your community heard about vaccines to protect against COVID-19?
4. **Concerns**:
a. What concerns do you, your family, or your community have about receiving a COVID-19 vaccine?
  i. If you have not heard anything that concerns you, what are some potential concerns you may have?
5. **Perceived risks/benefits:**
a. How do you think getting a COVID-19 vaccine would help you?
  1. Probe: How do you think you getting a COVID-19 might help others in your family? Your community?
b. What do you think are some risks of a COVID-19 vaccine?
6. **Perceived barriers/practical barriers/facilitators**
a. What are challenges you, your family, or people you know may face in getting the COVID-19 vaccine? Would anyone you know have difficulty obtaining the vaccine?
  i. Probe: what sorts of things may make it hard to get the COVID-19 vaccine? (personal health beliefs, fear of side effects, access to care, lack of trust, etc.)
7. **Trusted messengers**
a. People get information about COVID-19 in different ways. How do you get news and information about COVID-19? (Prompts: newspapers, TV, social media, family and friends, faith leader, your doctor or nurse)
b. Who do you trust the most to give you accurate information about COVID-19 and a COVID-19 vaccine?
8. **Health motivation**:
a. Do you think you will get the COVID-19 vaccine when it is available to the public?
  i. If yes, why?
  ii. If no, is there any new information or something else that might make you change your mind?
  iii. If you are on the fence, what information would you need to be comfortable in saying “yes” to getting the vaccine?
b. If you think you will get a COVID-19 vaccine when it is available, where would you feel most comfortable getting the vaccine? (doctor's office/clinic, your work site, public health department, pharmacy)
c. What could be done to make it easier for people who want to get a COVID-19 vaccine and need it most to get vaccinated when the vaccine is available?
9. **Take home message:** If there is someone you know who is not sure about getting a COVID-19 vaccine when it is available to the public, what would be the best way to help them make a decision about the vaccine that is right for them? (probe: one most important thing they should know)
10. Finally, is there anything else that you would like to add that we might not have asked you about? Do you have any questions?
